# A Novel Radiomics-based Interpretable Model for Bladder Cancer Grade Prediction Using White-Light Cystoscopy Images

**DOI:** 10.1016/j.euros.2026.03.018

**Published:** 2026-04-13

**Authors:** Yewon Choi, Sang Wouk Cho, Junho Hong, Jongsoo Lee, Hwiyoung Kim, Kwang Suk Lee

**Affiliations:** a Institute for Innovation in Digital Healthcare, Yonsei University, Seoul, South Korea; b Department of Integrative Medicine, Yonsei University College of Medicine, Seoul, South Korea; c Department of Biomedical Systems Informatics, Yonsei University College of Medicine, Seoul, South Korea; d Department of Artificial Intelligence, Yonsei University, Seoul, South Korea; e Department of Urology, Urological Science Institute, Severance Hospital, Yonsei University College of Medicine, Seoul, South Korea; f H-Data Strategy Center, Hallym University Medical Center, Chuncheon, Gangwon-do, South Korea; g Hallym University Chuncheon Sacred Heart Hospital, Chuncheon, Gangwon-do, South Korea; h Department of Urology, Urological Science Institute, Gangnam Severance Hospital, Yonsei University College of Medicine, Seoul, South Korea

**Keywords:** Cystoscopy, Diagnosis, Endoscopic procedure, Medicine, Surgery

## Abstract

**Background and objective:**

White-light cystoscopy (WLC) is the standard diagnostic modality for bladder cancer, but preoperative grading remains inaccurate. We developed a multichannel radiomics model to predict tumour grade (low-grade [LG] vs high-grade [HG]) from WLC and to identify imaging biomarkers.

**Methods:**

WLC images were retrospectively collected from 423 patients across two centres. A total of 2624 tumour regions were segmented for training, with 584 and 358 regions for internal and external validation, respectively. Radiomic features were extracted from the greyscale and red–green–blue channels. Feature selection was performed using coefficient thresholding and the least absolute shrinkage and selection operator. Five machine-learning classifiers were trained. Model performance was assessed using discrimination, calibration, and decision curve analysis (DCA). Interpretability was assessed using SHapley Additive exPlanations (SHAP) and feature visualisation.

**Key findings and limitations:**

The support vector machine model achieved robust performance, with an area under the receiver operating characteristic curve of 0.87 (95% confidence interval [CI] = 0.84–0.89) for internal validation and 0.79 (95% CI = 0.73–0.85) for external validation. SHAP analysis revealed distinct radiomic patterns differentiating LG from HG tumours. Limitations include retrospective design, manual segmentation, and a small, imbalanced external set, so validation reflects preliminary transportability rather than robustness or generalisability. Although calibration was acceptable and net benefit appeared at thresholds ≥ 0.30, external data constraints warrant caution.

**Conclusions and clinical implications:**

The proposed multichannel radiomics model supports grade prediction from WLC images and identifies a green channel. This approach provides a basis for developing real-time, filter-based tools for intraoperative risk stratification.

## Take Home Message

A novel radiomics model using cystoscopic images reliably predicts bladder-cancer grade and offers interpretable visual patterns, potentially reducing invasive procedures and accelerating diagnosis in clinical practice.

ADVANCING PRACTICE **What does this study add?**

We developed an explainable radiomics-based artificial intelligence (AI) model from white-light cystoscopy for bladder cancer grade classification. The model showed good performance across both internal and external validation. However, given the limited sample size, the external analysis was intended as a preliminary assessment of transportability. Interpretability was achieved through SHapley Additive exPlanations analysis and visual validation of key radiomic features.

**Clinical Relevance**

An interpretable radiomics-based AI model using standard white-light cystoscopy images can support preoperative prediction of bladder cancer grade, addressing the current limitations of subjective visual assessment.This approach may improve intraoperative decision-making during TURBT by enabling earlier risk stratification without additional imaging or invasive procedures. However, given the retrospective design and limited external validation, prospective studies are required before integration into routine clinical workflows. Associate Editor: Carmen Mir.

**Patient Summary**

We developed a machine-learning model that analyses bladder images obtained during routine endoscopy to predict tumour grade. This method may support faster and more accurate decision-making without requiring invasive biopsies, after further prospective validation.

## 1 Introduction

Bladder cancer (BCa) is the most common urinary tract malignancy, with approximately 600 000 new cases and over 200 000 deaths annually worldwide [1]. Accurate risk stratification at diagnosis is essential to guide treatment decisions and reduce the risk of recurrence or progression. High-grade (HG) tumours are associated with significantly worse outcomes than low-grade (LG) tumours, with progression and recurrence rates of 19% and 58%, respectively, compared to 4% and 43% for LG tumours [2]. As such, determining tumour grade prior to transurethral resection of bladder tumour (TURBT) is crucial for tailoring surgical extent and selecting appropriate adjuvant therapy [3], [4]. White-light cystoscopy (WLC) is currently the gold standard for evaluating BCa and guiding TURBT [5]. During cystoscopy, urologists visually assess tumour morphology, with HG lesions showing fuller papillary fronds and thicker stalks and LG lesions appearing thinner and more widely spaced, but this visual grading remains subjective and operator-dependent and achieves only moderate accuracy [6]. Despite the high sensitivity of WLC for detecting papillary lesions, it can still miss flat cancerous tissue or very small tumours, resulting in misdiagnosis rates of up to 30% [7], [8], [9]. Although repeat TURBT can improve diagnostic accuracy, it increases health care burden and procedural risks such as bladder perforation [10].

Recently, imaging-enhancement modalities such as blue light cystoscopy and narrowband imaging have been developed to improve tumour contrast. However, these methods remain subject to visual interpretation, leading to variability and diagnostic inconsistencies [5]. These limitations underscore the need for objective and reproducible methods to support tumour evaluation during cystoscopy. Artificial intelligence (AI) offers a promising solution to improve diagnostic consistency in cystoscopy, although most existing models focus on lesion detection or malignancy classification [11]. Systems like convolutional neural networks (CNN), CystoNet, and CAIDS have shown superior sensitivity and specificity to guide urologists in identifying lesions, including carcinoma in situ [12], [13]. Our team has also developed a WLC-based deep learning tool that detects suspicious bladder lesions with high sensitivity and shows promise as a clinical support tool [14].

Despite AI models demonstrating strong results in cystoscopic analysis, tumour grading before TURBT remains underdeveloped. Our previous study [15] showed that mean red–green–blue (RGB) intensity from WLC images could moderately distinguish benign or LG from HG BCa (area under the receiver operating characteristic curve [AUROC] = 0.70), but it lacked the ability to capture spatial and textural heterogeneity. Radiomics offers a more comprehensive analysis of tumour heterogeneity through quantitative imaging features [16]. However, to our knowledge, conventional radiomics has not been applied to endoscopic imaging. To address this gap, we developed a machine-learning (ML) framework that combines multichannel cystoscopy images with handcrafted radiomic features (HCRs), incorporating SHapley Additive exPlanations (SHAP) for interpretability. This approach enables stratification of pathological tumour grade in BCa and offers a basis for future development of a filter-based tool compatible with routine WLC workflows.

## 2 Patients and methods

### 2.1 Study design, patient selection, and data collection

**Fig. 1:**
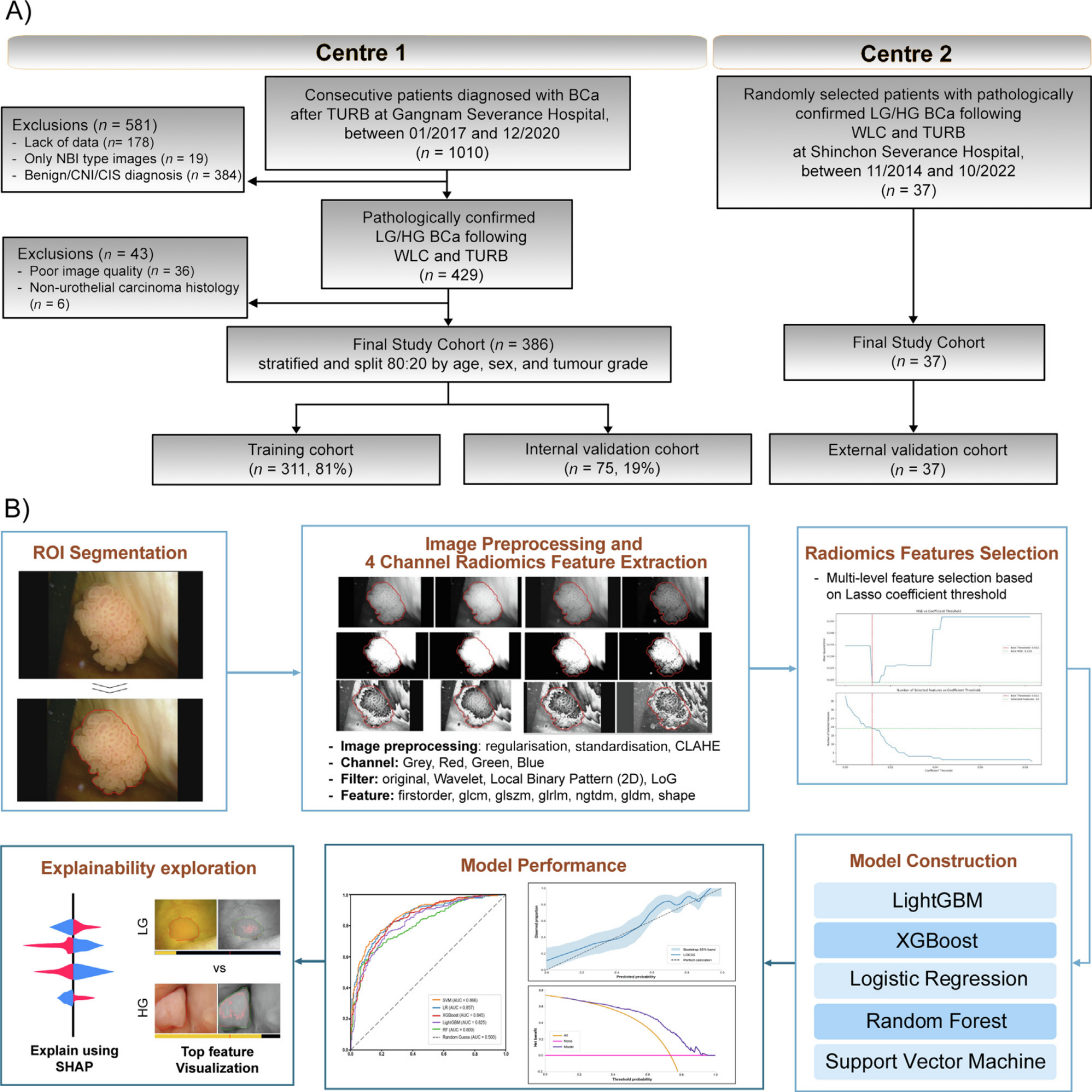
Overview of patient selection and radiomics-based BCa grade prediction workflow. (A) Flowchart of patient selection from two independent centres. Consecutive patients from Centre 1 and randomly selected eligible patients from Centre 2 were included based on pathologically confirmed LG/HG BCa following WLI cystoscopy and TURBT. The final cohort was stratified by age, sex, and tumour grade and split into training, internal validation, and external validation cohorts. (B) Development of a radiomics-based BCa grade prediction model. ROIs were manually segmented from cystoscopic images. Images were decomposed into four channels (grey, red, green, and blue) and preprocessed (regularisation, standardisation, and CLAHE). Radiomic features were extracted using the original, wavelet, local binary pattern, and Laplacian of Gaussian filters, followed by multistep feature selection based on the LASSO threshold. Five machine-learning classifiers were evaluated. Model performance was assessed using the AUROC and the AUPRC, calibration plots, and DCA. Feature contributions were interpreted using SHAP. LG = low-grade; HG = high-grade; BCa, bladder cancer; WLI = white-light imaging; TURBT = transurethral resection of bladder tumour; ROIs = regions of interest; CLAHE = contrast-limited adaptive histogram equalisation; AUROC = Area Under the Receiver Operating characteristic Curve; AUPRC = Area Under the Precision-Recall Curve; DCA = decision curve analysis; SHAP = SHapley Additive exPlanations.

This retrospective multicentre study was approved by the Institutional Review Boards (IRB) of Gangnam Severance Hospital (Centre 1, IRB No. 3-2023-0112) and Yonsei University Severance Hospital (Centre 2, IRB No. 1-2022-0052). The requirement for informed consent was waived by both institutions because of the use of fully anonymised data. The study adhered to the Declaration of Helsinki and was reported in accordance with the Transparent Reporting of a Multivariable Prediction Model for Individual Prognosis or Diagnosis with Artificial Intelligence (TRIPOD-AI) guidelines [17]. The workflow is illustrated in Fig. 1. Patient selection was independently performed at each centre (Fig. 1A). At Centre 1, 1010 consecutive patients who underwent TURBT between January 2017 and December 2020 were screened. Tumour grade (LG vs HG) was classified by board-certified pathologists with >15 yr of experience according to the 2004/2016 World Health Organization/International Society of Urological Pathology classification system and was used as the ground-truth label. Inclusion criteria were as follows: (1) histopathologically confirmed urothelial carcinoma with LG or HG grade, and (2) availability of preoperative WLC images suitable for analysis. Exclusion criteria were as follows: (1) lack of clinical or imaging data, (2) poor-quality WLC images (eg, presence of stones within the bladder lesion, turbid urine, haematuria, images that were too dark or blurry to interpret, or out-of-focus imaging), and (3) nonurothelial carcinoma histology. After applying these criteria, 386 patients were included in the study. An 8:2 stratified split based on age, sex, and tumour grade was used to divide the patients into training (*n* = 311) and internal validation (*n* = 75) cohorts. An external validation cohort of 37 patients from Centre 2 was selected using the same criteria. Cohort allocation was performed at the patient level, and all tumour regions from each patient were assigned to the same cohort and used as individual units for radiomic feature extraction and model evaluation. These patients underwent WLC and TURBT between November 2014 and October 2022.

### 2.2 Image acquisition and tumour segmentation

All patients underwent WLC with a flexible cystoscope (CYF-V2; Olympus Medical Systems Corporation, Tokyo, Japan). Tumour regions were manually segmented from the WLC images to augment the training volume. In total, 2624 tumour regions were segmented from the training cohort, 584 from the internal validation cohort, and 358 from the external validation cohort. Manual segmentation was independently performed at each centre by experienced urologists (>10 yr of TURBT experience) using a standardised annotation protocol and identical software (ITK-SNAP, version 3.8.0). At Centre 1, preannotated data from Yoo et al. [15] were used for segmentation, whereas Centre 2 followed the same protocol independently. The annotators were blinded to clinical and pathological data.

### 2.3 Radiomic feature extraction and selection

Radiomic features were extracted from the segmented regions following standardised preprocessing (Fig. 1B). Images were decomposed into four colour channels (red, green, blue, and greyscale) and processed using regularisation, standardisation, and contrast-limited adaptive histogram equalisation. Feature selection was performed using the Coe-Thr-Lasso algorithm [18], a radiomics-oriented method that integrates variance filtering, Welch’s *t*-test, and recursive Lasso regularisation. Proven effective in radiomics studies, this multilevel approach identifies a compact, nonredundant feature subset that enhances model generalisability. For comparison, Lasso, recursive feature elimination with cross-validation, and random forest (RF)-importance methods were also evaluated (Supplementary Table 1). The Coe-Thr-Lasso algorithm process reduced the initial features to 19 optimal predictors using a threshold of 0.012, yielding a minimum loss of 0.219 during model optimisation (Supplementary Section S1 and Supplementary Figure 1). Detailed preprocessing steps, filtering parameters, feature selection thresholds, and feature description are provided in Supplementary Section S1 and Supplementary Table 3.

### 2.4 Radiomics model construction and explanation

The selected 19 radiomic features were used to train five classifiers spanning three complementary modelling paradigms: (1) a linear model (logistic regression, LR) used solely as a baseline comparator, (2) a kernel-based nonlinear model (support vector machine with a radial basis function kernel, SVM [support vector machine]), and (3) tree-based ensemble models (RF, XGBoost, and LightGBM) used to flexibly capture nonlinear effects and feature interactions.

Model development was conducted using Centre 1 training data with stratified five-fold cross-validation and hyperparameter tuning using the Optuna framework [19]. Class imbalance was addressed by applying weighted loss functions. Details of hyperparameter optimisation are provided in Supplementary Section S2. Trained models were evaluated on internal (Centre 1) and external (Centre 2) validation cohorts without retraining to assess generalisability. Each model produced continuous probability scores (range = 0–1) for HG classification. SHAP, a game theory–based model interpretation method [20], was used to explain predictions of the machine-learning model. The most influential radiomic feature was visualised using a standardised image-processing pipeline (Supplementary Fig. 1) to illustrate its spatial origin, and these visualisations were provided solely for post hoc interpretability.

### 2.5 Statistical analysis

The statistical analysis followed published guidelines [21]. Baseline patient characteristics were summarised to describe the study cohort using median (interquartile range [IQR]) for continuous variables and number (%) for categorical variables (Table 1). Radiomics features were extracted and analysed at the tumour-region level, with multiple regions per patient. To prevent data leakage, cohort allocation was performed at the patient level so that all regions from the same patient were assigned to the same cohort. Model discrimination was evaluated using the AUROC and the area under the precision–recall curve (AUPRC) to assess the ability of the models to distinguish HG from LG tumour regions. Differences in discrimination performance were compared using the DeLong test [22]. Model calibration was evaluated using locally estimated scatterplot smoothing line-smoothed calibration plots comparing predicted probabilities with observed outcome frequencies, whereas clinical utility was assessed using decision curve analysis (DCA). These analyses were performed in the external validation set. The 95% confidence intervals (CI) were estimated using a patient-level cluster bootstrap (2000 resamples), resampling patient IDs with replacement and including all regions per resampled patient to account for within-patient clustering [23]. Radiomic features were extracted using Pyradiomics version 3.0.1 [24]. All analyses were conducted using R version 4.0.4 and Python version 3.9.7. Statistical significance was defined as *p* < 0.05.

**Table 1:** Patient characteristics

Characteristics	Training cohort	Internal validation cohort	External validation cohort
	Centre 1	Centre 1	Centre 2
Patients, *n*	311	75	37
Male gender, *n* (%)	261 (84)	60 (80)	35 (95)
Age (yr), median (IQR)	69 (61–76)	68 (58–78)	70 (60.0–77)
Tumour region, *n*	2624	584	358
HG, *n* (%)	1512 (58)	329 (56)	262 (74)

SD = standard deviation; HG = high grade; LG = low grade.

## 3 Results

### 3.1 Patient characteristics

Mean age and sex distribution were similar across the training, internal validation, and external validation cohorts (Table 1). From WLC images of each cohort, 2624, 584, and 358 tumour regions were segmented, respectively. The proportion of HG was similar between the training and internal validation cohorts, whereas a higher proportion (74%) was observed in the external cohort compared with the other groups.

### 3.2 Feature selection

The final 19 radiomic features extracted from the RGB and greyscale channels were selected (Supplementary Tables 2 and 3), including six first-order statistics and 13 texture features. The selected features were distributed across different colour channels: green (*n* = 6), red (*n* = 6), blue (*n* = 4), and greyscale (*n* = 3). Regarding filtering methods, log-sigma–filtered features comprised the largest proportion (*n* = 10), followed by the original features (*n* = 5) and wavelet-transformed features (*n* = 4). This distribution indicates that features derived from multiple colour channels and various filtering approaches contributed to the final predictive model.

### 3.3 Development and testing of radiomics-based ML model

Five ML classifiers were evaluated, and the SVM demonstrated the best performance (Supplementary Fig. 2). In internal validation, the SVM achieved an AUROC of 0.87 (95% CI = 0.84–0.89) and an AUPRC of 0.89 (95% CI = 0.86–0.92) (Table 2). In external validation, discrimination was maintained (AUROC, 0.79 [95% CI = 0.73–0.85]; AUPRC, 0.90 [95% CI = 0.85–0.93]).

**Table 2:** Diagnostic performance of radiomics-based machine learning models in internal and external validation cohorts^a^

	AUROC (95% CI)	AUPRC (95% CI)
Internal validation cohort		
SVM	0.87 (0.81–0.91)	0.89 (0.81–0.95)
LR	0.86 (0.80–0.90)	0.89 (0.81–0.94)
XGBoost	0.85 (0.78–0.89)	0.87 (0.78–0.93)
LightGBM	0.83 (0.76–0.88)	0.85 (0.76–0.91)
RF	0.84 (0.77–0.89)	0.85 (0.78–0.93)
External validation cohort		
SVM	0.79 (0.71–0.88)	0.90 (0.80–0.96)
LR	0.79 (0.71–0.88)	0.89 (0.79–0.96)
XGBoost	0.73 (0.64–0.85)	0.85 (0.73–0.93)
LightGBM	0.58 (0.58–0.71)	0.79 (0.67–0.93)
RF	0.68 (0.63–0.77)	0.85 (0.73–0.93)

AUROC = area under the receiver operating curve, AUPRC = area under the precision-recall curve, LR = logistic regression, RF = random forest, SVM = support vector machine CI = confidence interval.

a Performance is reported at the tumour region level, and the 95% CIs were estimated using patient-level cluster bootstrap resampling (2000 iterations) to account for within-patient clustering.

Model calibration and clinical utility in the external validation cohort are summarised in Fig. 3. Calibration demonstrated acceptable agreement between predicted and observed risk (intercept 0.43 [95% CI = 0.10–0.76]; slope 0.93 [95% CI = 0.67–1.18]), although the CI was wide. DCA showed a net benefit of the SVM over treat-all and treat-none for threshold probabilities ≥ 0.30. At this threshold, the implied trade-off corresponds to accepting approximately 2.3 unnecessary escalations to HG management per additional true HG region detected, indicating clinical utility within moderate-risk decision contexts.

### 3.4 Exploration of model interpretability

**Fig. 2:**
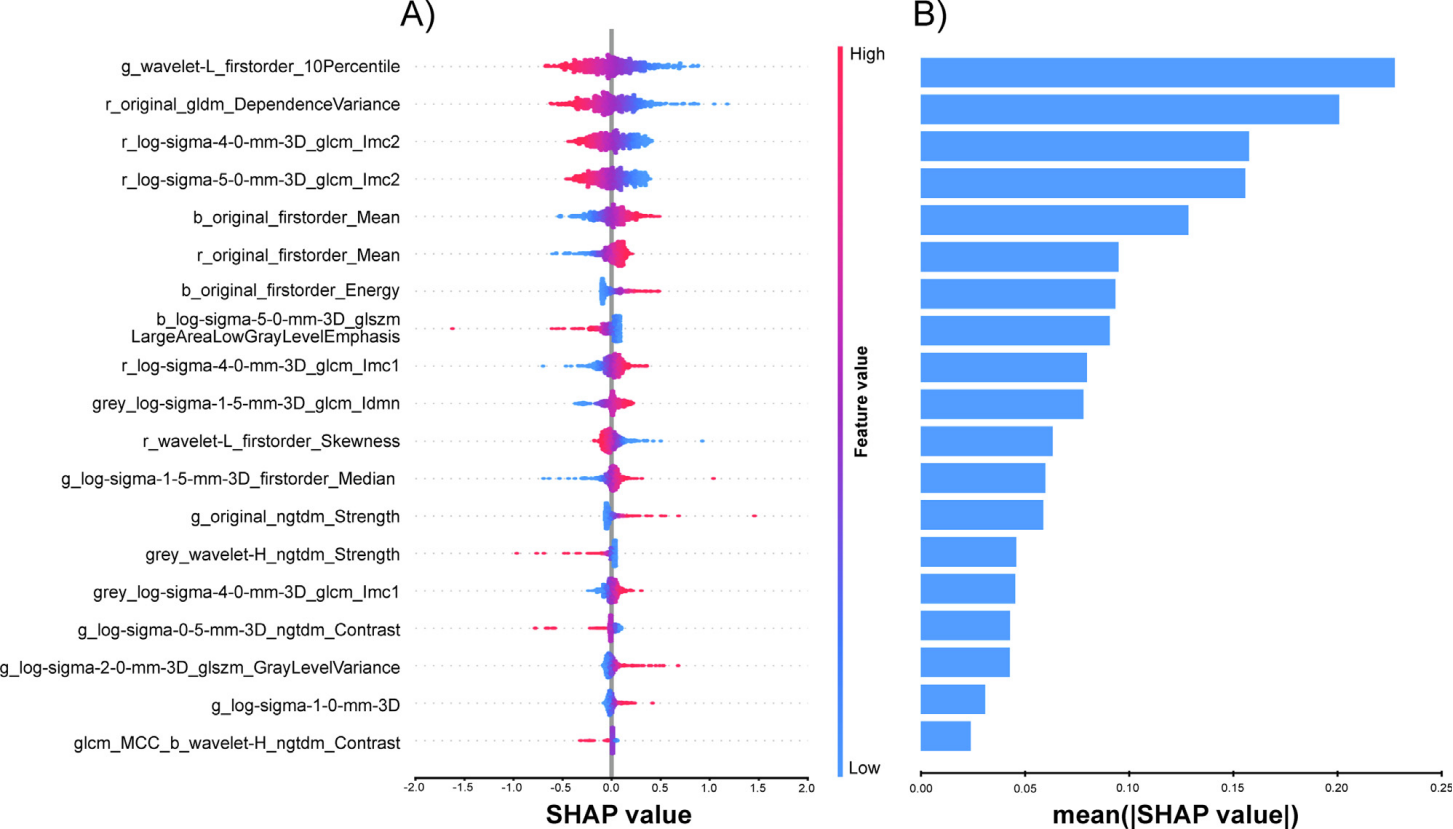
Global visualisation of the SVM model through SHAP. (A) Beeswarm plot showing the distribution of SHAP values for each feature across tumour regions. Point colour denotes the feature value (red, high; blue, low), and the x-axis indicates the direction and magnitude of each feature’s contribution to predicting HG (positive) versus LG (negative). (B) Bar plot of mean absolute SHAP values, summarising each feature’s global importance. Features are denoted using the concatenated format [image channel]_[filter]_[feature class]_[feature name]. [image channel] indicates r, g, b, or grey; [filter] indicates original (unfiltered), wavelet, or LoG; and [feature_class] includes first-order statistics and texture-based classes (e.g., GLCM, GLSZM, GLDM and NGTDM). SHAP = SHapley Additive exPlanations; red = r; green = g; blue = b; greyscale = grey; Laplacian of Gaussian = LoG; GLCM = Grey-Level Co-occurrence Matrix; GLDM = Grey-Level Dependence Matrix; GLSZM = Grey-Level Size Zone Matrix; NGTDM = Neighbouring Grey Tone Difference Matrix; IMC = Informational Measure of Correlation.

**Fig. 3:**
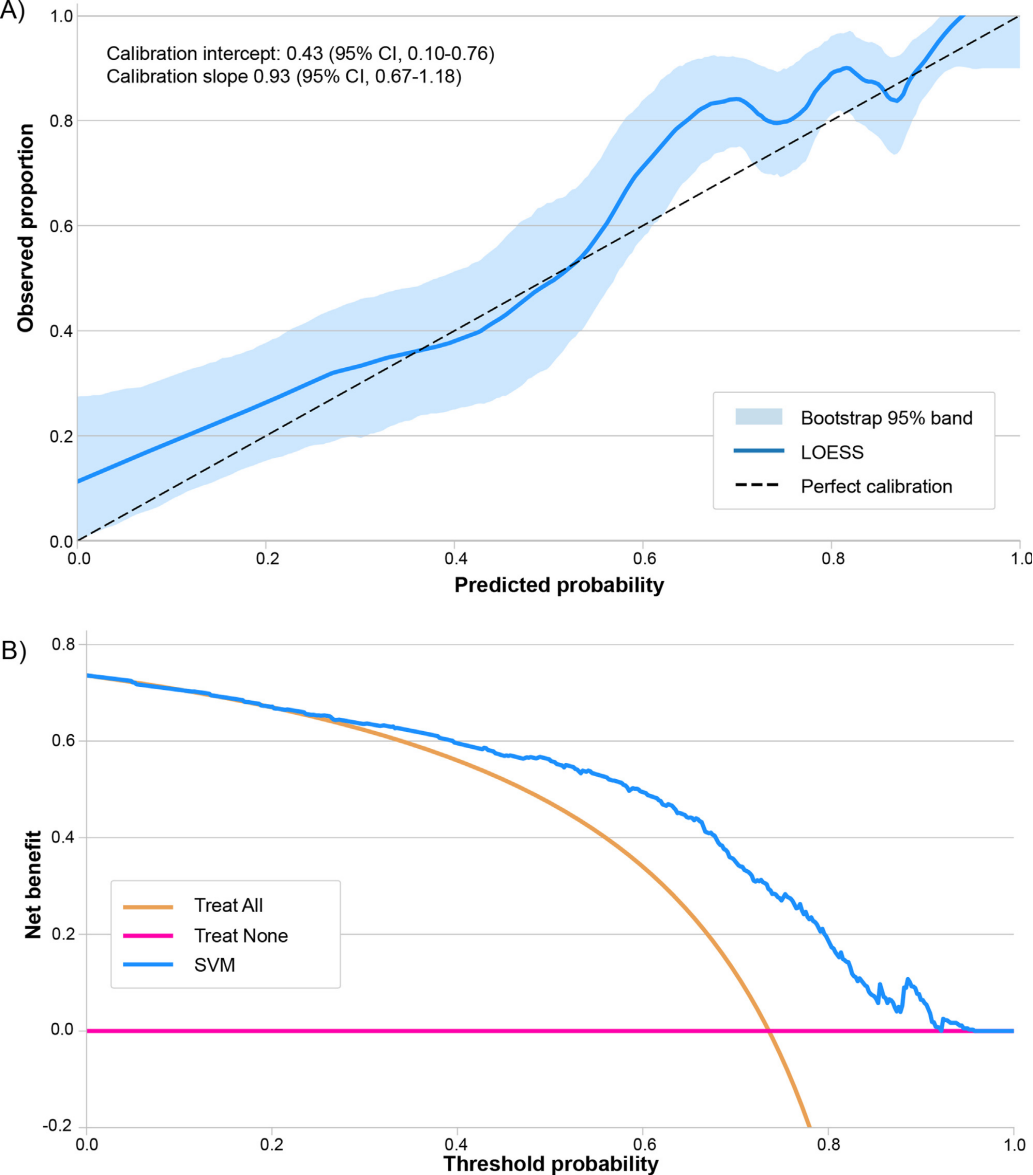
Calibration and decision curve analysis of the SVM model for predicting high-grade bladder tumour regions. (A) Calibration plot. The LOESS-smoothed curve (solid blue) depicts agreement between predicted probabilities and observed proportions; the shaded band represents the bootstrap-derived 95% CI. The dashed diagonal line indicates perfect calibration. (B) Decision curve analysis. Net benefit across threshold probabilities is shown for the SVM-guided classification (purple), “treat-all” (orange; all tumour regions managed as high grade), and “treat-none” (pink; all tumour regions managed as low grade) strategies. LOESS = locally estimated scatterplot smoothing line; DCA = Decision curve analysis; CI = confidence interval; SVM = Support Vector Machine.

We calculated SHAP values for each radiomics feature in the SVM model. In the global interpretation, the SHAP bar plot (Fig. 2B) showed the relative importance of each feature. The wavelet-L-filtered 10th percentile feature from the green channel (g_wavelet-L_firstorder_10Percentile, GWL10) contributed most to the SVM model. We also generated a SHAP summary (beeswarm) plot (Fig. 2A) to illustrate the impact of each feature on the model predictions, showing that lower feature values consistently contributed towards HG classification, whereas higher feature values contributed toward LG classification. This directionality was further supported by the negative LR coefficient for GWL10 in the training cohort (Supplementary Table 2), indicating that lower GWL10 consistently contributed to HG classification. When visualised post hoc using the standardised image-processing pipeline (Supplementary Fig. 1), the pixels contributing to the 10th-percentile value in cases predicted as LG showed a scattered and heterogeneous spatial distribution with diffuse expression across the tumour region. In contrast, cases predicted as HG were associated with more concentrated and organised clusters of such low-intensity pixels, often exhibiting a peripheral rim-like distribution. These spatial expression patterns were consistently observed across both correctly and incorrectly classified cases, as illustrated in representative examples from internal and external validation cohorts (Fig. 3) and extended examples (Supplementary Figs. 3 and 4).

## 4 Discussion

This study showed HCRs extracted from WLC images can predict pathological grade in BCa. Among the evaluated classifiers, the SVM achieved the highest internal discrimination and maintained discrimination in the external cohort. Model performance was systematically evaluated through SHAP analysis for interpretability and feature visualisation. Calibration and DCA provided exploratory insights into potential net benefit.

Accurate preoperative grading of BCa is crucial for determining tumour invasion and optimal treatment [25], [26]. Distinguishing LG and HG tumours before TURBT offers meaningful benefits by guiding surgical planning and reducing over- or undertreatment. Currently, BCa grade is determined by cystoscopy and TURBT histology [27], but accuracy may be limited by sampling and operator variability. A noninvasive, image-based tool providing accurate intraoperative risk stratification could help address this unmet need.

Most prior studies on pathological grade prediction in BCa have focused on computed tomography, magnetic resonance imaging, or ultrasound-based radiomics models [28], [29], [30], [31], [32], with AUROC values of 0.73–0.95. While these modalities effectively assess muscle invasion and metastatic disease, they often miss small or flat early-stage lesions and present limitations, including high cost, radiation exposure, and limited accessibility. In contrast, cystoscopy is routine in clinical practice and enables real-time visualisation. Only a few AI models have addressed grade classification using cystoscopic images. The colour-based approach proposed by Yoo et al. [15] lacked the feature richness and analytic depth required for stable grade discrimination. Ali et al. [33] developed a blue light imaging-based CNN that showed high sensitivity and specificity, but its generalisability was limited by a small dataset and dependence on nonroutine imaging. Our approach advances prior work by using a radiomics framework that captures spatial and textural heterogeneity, applies an effective feature-selection method to identify key predictors, incorporates SHAP-based interpretability, and is externally validated with routine WLC images, the clinical gold standard. Although the AUROC values were modest, the model outperformed our prior mean RGB intensity approach (AUROC = 0.70) [15]. Our SVM showed incremental net benefit over treat-all strategies at thresholds ≥0.30, suggesting potential utility within moderate-risk decision contexts. The lack of benefit at lower thresholds likely reflects the high HG prevalence and small sample size of the cohort, which inherently favours treat-all strategies in DCA and supports validation in more heterogeneous populations [34].

To our knowledge, this is the first application of HCRs to routine cystoscopy in BCa, and the framework facilitates exploration of image-derived patterns associated with tumour grade.

Feature selection using the Coe-Thr-Lasso algorithm [18] identified 19 key predictors, notably excluding shape features. This finding is consistent with previous studies [28], [30] suggesting that, for BCa grading, texture features are more informative than shape features. As intratumoural heterogeneity, captured by texture features, has been associated with higher tumour grade [35], its predominance highlights the value of texture features for grade discrimination. In both our SVM and LR models, GWL10 emerged as the most influential feature for grade classification (Fig. 2B, Supplementary Table 2). This is consistent with the observed directionality and with prior reports that green-channel imaging enhances vascular contrast due to haemoglobin absorption [36] and that wavelet transforms effectively capture intratumoural heterogeneity [37].

Although SHAP values quantify feature importance, the abstract nature of radiomic descriptors limits clinical interpretability. We therefore used post hoc GWL10 visualisation to spatially map dominant radiomic signals onto WLC images. This analysis is exploratory and explanatory, serving as an intermediate step toward future clinically deployable filtering or decision-support frameworks. Prospective studies are required to assess whether such feature-driven visual cues improve real-time cystoscopic decision-making. In the absence of histopathological correlation, the observed signal patterns should be considered preliminary and require validation against pathological substrates (eg, microvascular density and cellular atypia).

Our study has some limitations. First, the modest AUROC likely reflects the inherent limitations of WLC as a standalone modality for tumour grade discrimination. Nonetheless, model performance was broadly comparable to the precision reported for expert urologists by Mariappan et al. [6] (85.8% for LG and 71.3% for HG), although the metrics are not directly matched. Integration of additional imaging or clinical variables may further enhance performance. Second, analyses were conducted at the tumour-region level without explicit hierarchical modelling of regions nested within patients; although patient-level cluster bootstrapping was applied, residual within-patient correlation may affect precision estimates. Third, intraoperative deployment and workflow integration were not evaluated, precluding comparison with real-time surgeon grading or assessment of impact on TURBT-related decision-making. Fourth, the external validation cohort was smaller than recommended for models with an AUROC > 0.80 [38] (Supplementary Section 3) and was grade imbalanced. The external cohort was included to explore signal persistence across centres rather than to establish model robustness or clinical readiness. Accordingly, overall model performance, including discrimination, calibration, and DCA should be interpreted as a preliminary assessment of transportability rather than definitive evidence of robustness or generalisability. Finally, manual segmentation may introduce inter-observer variability, limiting reproducibility and scalability. Future work should include larger prospective multicentre cohorts to confirm model stability and effectiveness in real-world settings and to determine whether the approach influences intraoperative decision-making, including the extent or timing of TURBT. Integration of automated lesion detection and segmentation into an AI-assisted cystoscopy workflow may facilitate future clinical implementation.

## 5 Conclusions

Our radiomics-based ML model using WLC images predicted BCa grade with good internal discrimination and similar performance in a small external cohort, indicating preliminary transportability. Calibration was acceptable, and DCA indicated potential clinical utility at thresholds ≥0.30. SHAP identified GWL10 as a key, interpretable contributor linking image heterogeneity to tumour grade. Our approach supports a complementary, noninvasive decision-support framework with real-time applicability. However, larger prospective and real-world validations are needed, given the retrospective design, manual segmentation, and limited external cohort size.

  ***Author contributions****:* Kwang Suk Lee had full access to all the data in the study and takes responsibility for the integrity of the data and the accuracy of the data analysis.

  *Study concept and design:* Choi, Cho, K. Lee.

*Acquisition of data:* J. Lee, K. Lee.

*Analysis and interpretation of data:* Choi, Cho, J. Lee, K. Lee.

*Drafting of the manuscript:* Choi, Cho, K. Lee.

*Critical revision of the manuscript for important intellectual content:* Choi, Cho, Kim, K. Lee.

*Statistical analysis:* Choi, Cho, Hong.

*Obtaining funding:* None.

*Administrative, technical, or material support:* Choi, Cho, K. Lee.

*Supervision:* Kim, K. Lee.

*Other:* None.

  ***Financial disclosures:*** Hwiyoung Kim and Kwang Suk Lee certify that all conflicts of interest, including specific financial interests and relationships and affiliations relevant to the subject matter or materials discussed in the manuscript (eg, employment/affiliation, grants or funding, consultancies, honoraria, stock ownership or options, expert testimony, royalties, or patents filed, received, or pending), are the following: None.

  ***Funding/Support and role of the sponsor:*** This study was supported by a faculty research grant of Yonsei University College of Medicine (6-2023-0236) and the Technology Innovation Program (RS-2025-02220286, Development of large language AI model-based techniques and platforms for nursery record generation and task automation) funded by the Ministry of Trade, Industry & Resources (MOTIR, Korea).

  ***Acknowledgements*:** We would like to thank Editage (www.editage.co.kr) for English language editing.
