# Supplementary material for: A Novel Radiomics-based Interpretable Model for Bladder Cancer Grade Prediction Using White-Light Cystoscopy Images
Source: Eur Urol Open Sci. 2026 Apr 13;87:71–9. doi: 10.1016/j.euros.2026.03.018 (PMC13094432; doi:10.1016/j.euros.2026.03.018)
Supplement: Supplementary Data 1 [file mmc1.docx]

**Supplementary material**

**Section S1. Feature extraction and selection protocol**

**Section S2. Details on model development**

**Section S3. Sample-size Calculation for Internal and External Validation**

**Section S4. Supplementary table**

**Supplementary Table 1.** Performance comparison of radiomics-based classifiers with different feature selection algorithms

**Supplementary Table 2.** Logistic regression coefficients of selected radiomic features

**Supplementary Table 3.**Definitions for Selected Radiomics Feature

**Section S5. Supplementary figures**

**Supplementary Figure 1.** Systematic preprocessing pipeline for visualisation of the highest-impact radiomics feature (g_wavelet-L_firstorder_10Percentile)

**Supplementary Figure 2.** Performance comparison of radiomics-based classifiers

**Supplementary Figure 3.** Analysis of g_wavelet-L First-Order 10th Percentile features between correctly and incorrectly classified bladder cancer grades in the internal validation cohort

**Supplementary Figure 4.** Analysis of g_wavelet-L First-Order 10th Percentile features between correctly and incorrectly classified bladder cancer grades in the external validation cohort

**Section S1. Feature extraction and selection protocol**

Feature selection was performed to reduce feature redundancy and limit model complexity. Using the Coe-Thr-Lasso approach, a coefficient threshold of θ = 0.012 minimized loss while reducing the feature set to 19 features (details in Section S1 Figure 1).

**Feature extraction methodology (total extracted features [n=4,473]):**

A. Channel-specific features (n=4,464)

1. Extraction from all colour channels (red, green, blue, greyscale)
2. Applied filters: original, wavelet, LBP (2D), LoG
3. Features per channel: 1,116 (279 features × 4 filters)
4. Feature types: first-order statistics and texture features (GLCM, GLSZM, and GLRLM)

B. Shape features (n=9)

1. Extracted exclusively from original greyscale images
2. No additional filtering applied

**Feature selection results**

1. Variance filtering: 4,473 → 4,294
2. Statistical testing (p < 0.05): 4,294 → 3,097
3. Lasso optimisation (θ=0.012): 3,097 → 19
4. Model performance: minimum loss=0.219

**
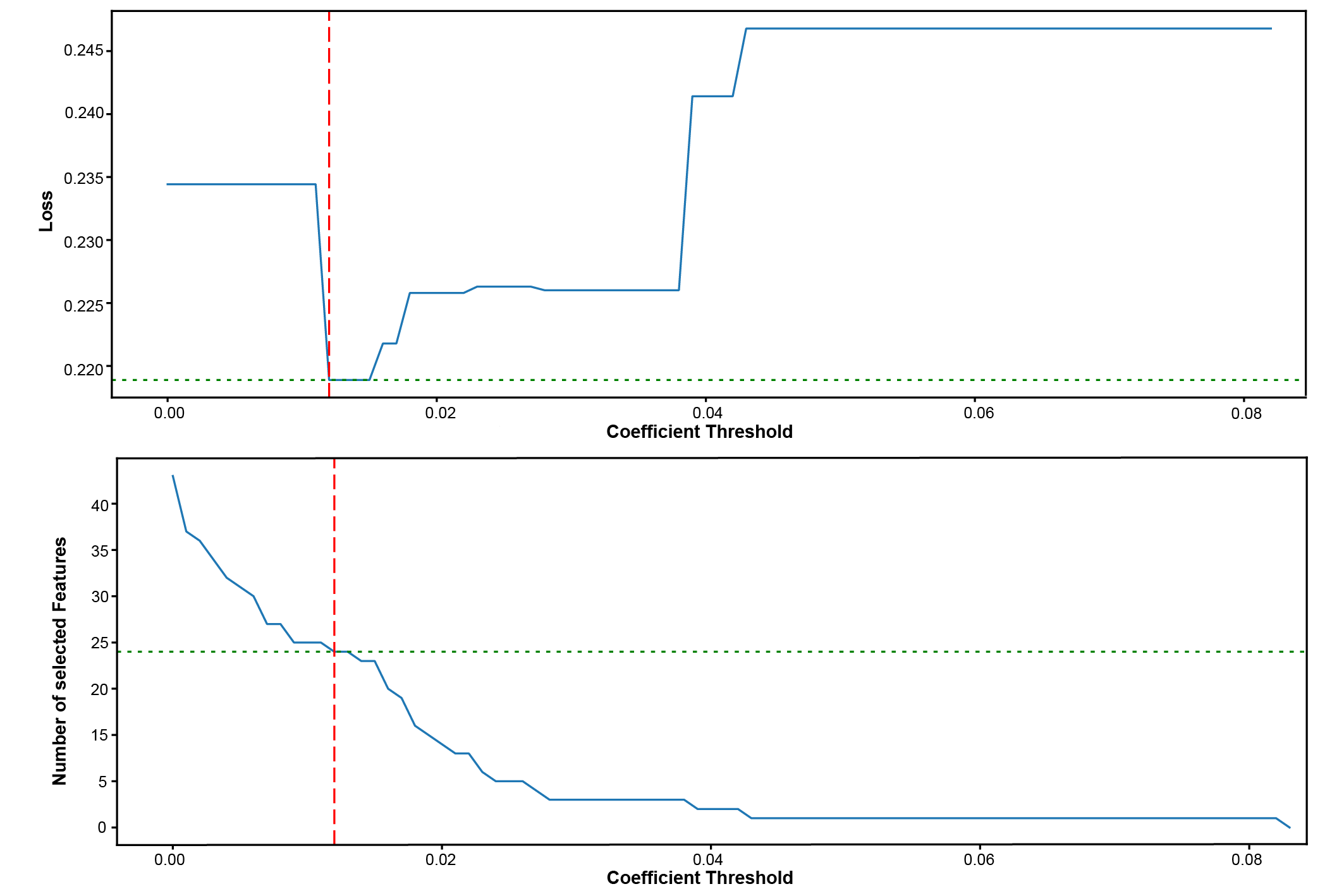
**

**Section S1 Figure 1. Feature selection optimisation using the Coe-Thr-Lasso algorithm**

The figure illustrates the optimisation of the lasso coefficient threshold proposed by Wang et al. The upper panel shows the model loss across coefficient thresholds, and the lower panel shows the corresponding number of selected features. The selected threshold (θ = 0.012, red dashed line) corresponds to the minimum loss (0.219) while reducing the feature set to 19 features, illustrating the trade-off between model fit and feature sparsity.

**Section S2. Details on model development**

1. LR

penalty='l2', dual=False, tol=0.0001, C=1.327, fit_intercept=True, intercept_scaling=1, class_weight={0: 1.180, 1:0.868}, random_state=42, solver='liblinear', max_iter=1000, multi_class='auto', verbose=0, warm_start=False, n_jobs=None, l1_ratio=None.

2. SVM

C=2584.591, kernel='rbf', degree=3, gamma=0.167e-3, coef0=0.0, shrinking=True, probability=True, tol=0.001, cache_size=200, class_weight={0: 1.180, 1: 0.868}, verbose=False, max_iter=-1, decision_function_shape='ovr', break_ties=False, random_state=42.

3. RF

n_estimators=755, criterion='gini', max_depth=28, min_samples_split=15, min_samples_leaf=1, min_weight_fraction_leaf=0.0, max_features=0.212, max_leaf_nodes=None, min_impurity_decrease=0.0, bootstrap=True, oob_score=False, n_jobs=-1, random_state=42, verbose=0, warm_start=False, class_weight={0: 1.180, 1: 0.868}, ccp_alpha=0.0, max_samples=None.

4. XGBoost

boosting_type='gbdt', learning_rate=0.383, max_depth=14, min_child_samples=12, min_child_weight=0.001, min_split_gain=0.0, n_estimators=1349, num_leaves=1023, subsample=1.0, colsample_bytree=0.500, reg_lambda=14.074, random_state=42, scale_pos_weight=0.735, early_stopping_rounds=20, verbose=-1.

5. LightGBM

boosting_type='gbdt', learning_rate=0.383, max_depth=14, min_child_samples=12, min_child_weight=0.001, min_split_gain=0.0, n_estimators=1349, num_leaves=1023, subsample=1.0, colsample_bytree=0.500, reg_lambda=14.074, random_state=42, scale_pos_weight=0.735, early_stopping_rounds=20, verbose=-1.

**Section S3. Sample-size calculation for internal and external Validation**

To assess whether our internal and external validation cohorts (n=584 and n=358 tumour regions, respectively) provided sufficient statistical power to evaluate the discriminatory performance of our radiomics-based prediction model, we performed a sample-size calculation using the pmsampsize package (version 1.1.3) in R. This method, proposed by Riley et al. (BMJ, 2024)) [[1]](https://www.zotero.org/google-docs/?XKS6Bp), provides a structured framework for estimating the minimum sample size required for external validation studies based on the expected area under the curve (AUROC), number of model parameters, and prevalence of the outcome.

We assumed an expected AUROC of **0.80**, which represents the acceptable discriminatory ability for clinical prediction models [2]. The final model contained **19 predictors**, and the prevalence of high-grade (HG) tumours was 58**%**, which was consistent across our training cohort (1512/2624) and prior population-based pathology studies in Korean NMIBC patients [3].

The following R code was used for the sample-size calculation:

| library(pmsampsize)    result <- pmsampsize(  type = "b",  cstatistic = 0.80, # anticipated AUC  parameters = 19, # Number of predictors  prevalence = 0.58 # prevalence of high-grade tumours in korea    print(result) |
| --- |

Based on these parameters, the minimum required sample size for reliable validation was estimated to be **568 tumour regions**, including at least **330 HG events**. Our internal validation dataset (584 tumour regions, 329 HG events) almost met this requirement, confirming adequate power for internal validation. In contrast, the external validation dataset included 358 tumour regions and 262 HG events while the total sample size fell slightly short, the number of events exceeded the recommended threshold.

1. Riley RD, Snell KIE, Archer L, et al. Evaluation of clinical prediction models (part 3): calculating the sample size required for an external validation study. BMJ 2024;384:e074821.

2. Mandrekar JN. Receiver operating characteristic curve in diagnostic test assessment. J Thorac Oncol 2010;5:1315-6.

3. Kim JY, Lee DB, Song WH, Lee SS, Park SW, Nam JK. External validation of European Association of Urology NMIBC risk scores to predict progression after transurethral resection of bladder tumour in Korean patients with non-muscle-invasive bladder cancer. Investig Clin Urol 2022;63:531-8.

**Section S4. Supplementary Tables**

**Supplementary Table 1.** Performance comparison of radiomics-based classifiers with different feature selection algorithms

| Methods | Feature Count | LR | | RF | | SVM |  |
| --- | --- | --- | --- | --- | --- | --- | --- |
|  |  | AUROC | AUPRC | AUROC | AUPRC | AUROC | AUPRC |
| Lasso | 21 | 0.84 | 0.85 | 0.83 | 0.86 | 0.84 | 0.87 |
| RFECV | 21 | 0.83 | 0.85 | 0.80 | 0.84 | 0.85 | 0.88 |
| RF Importance | 12 | 0.82 | 0.84 | 0.80 | 0.84 | 0.81 | 0.84 |
| Coe-ThrLasso | 19 | 0.86 | 0.89 | 0.81 | 0.85 | 0.87 | 0.89 |

LR = logistic regression, RF = random forest, SVM = support vector machine, RFECV = recursive feature elimination with cross-validation, AUROC = area under the receiver operating characteristic, AUPRC = area under the precision-recall curve.

**Supplementary Table 2.** Logistic regression coefficients of selected radiomic features

| **Logistic Regression Model Feature** | **Coefficients** |
| --- | --- |
|  |  |
| g_wavelet-L_firstorder_10Percentile | -0.250 |
| r_original_gldm_DependenceVariance | -0.189 |
| r_original_firstorder_Mean | 0.173 |
| b_original_firstorder_Mean | 0.171 |
| g_original_ngtdm_Strength | 0.167 |
| r_log-sigma-5-0-mm-3D_glcm_Imc2 | -0.161 |
| grey_wavelet-H_ngtdm_Strength | -0.157 |
| r_log-sigma-4-0-mm-3D_glcm_Imc2 | -0.156 |
| b_log-sigma-5-0-mm-3D_glszm_LargeAreaLowGreyLevelEmphasis | -0.142 |
| grey_log-sigma-1-5-mm-3D_glcm_Idmn | 0.131 |
| g_log-sigma-1-5-mm-3D_firstorder_Median | 0.130 |
| r_wavelet-L_firstorder_Skewness | -0.130 |
| g_log-sigma-0-5-mm-3D_ngtdm_Contrast | -0.118 |
| r_log-sigma-4-0-mm-3D_glcm_Imc1 | 0.118 |
| b_original_firstorder_Energy | 0.081 |
| b_wavelet-H_ngtdm_Contrast | -0.078 |
| grey_log-sigma-4-0-mm-3D_glcm_Imc1 | 0.078 |
| g_log-sigma-1-0-mm-3D_glcm_MCC | 0.066 |
| g_log-sigma-2-0-mm-3D_glszm_GreyLevelVariance | 0.052 |

Note: Features are denoted using a concatenated format [image_channel]_[filter]_[feature_group]_[feature_name]. For instance, g_wavelet-L_firstorder_10Percentile represents a feature extracted from the green channel (g) after applying a wavelet-L filter, belonging to the first-order statistical group, specifically measuring the 10th percentile value. Logistic regression coefficients are provided to quantify the direction and magnitude of each feature’s association with low-grade (vs high-grade) prediction.

**Supplementary Table 3.** Definitions for Selected Radiomics Feature

| **Feature category** | | **Description** | **Feature** | **Explanation** |
| --- | --- | --- | --- | --- |
| First-order | | Describes the distribution of voxel intensities within the tumour | 10Percentile | The intensity level below which 10% of pixels fall, highlighting darker parts within the tissue. |
|  |  |  | Mean | Shows the average brightness, providing an idea of general tissue density in the tumor. |
|  |  |  | Median | The middle brightness value, giving a sense of how "bright" or "dark" the overall tumor area is. |
|  |  |  | Skewness | Reflects the asymmetry of intensity values, indicating a shift towards either brighter or darker regions within the tissue. |
|  |  |  | Energy | Measures the magnitude/sum of squared pixel intensities. |
| Texture (13) | GLCM | Describes the second-order joint probability function of the tumour | Imc1 | Measures the degree of structured texture by quantifying pixel intensity correlations. |
|  |  |  | Imc2 | Captures complex spatial relationships and texture dependencies at a larger scale. |
|  |  |  | Idmn | Measures local uniformity; higher values mean neighboring areas have similar intensity, indicating smooth texture. |
|  |  |  | MCC | Reflects intermediate-scale texture complexity based on pixel co-occurrence patterns. |
|  | GLSZM | Quantifies grey-level zones in the tumour | LargeAreaLowGreyLevelEmphasis | Highlights large, darker areas within the tumor, which could correspond to necrotic or less active tissue regions. |
|  |  |  | GreyLevelVariance | Measures intensity variability across homogeneous zones, reflecting intratumoural heterogeneity. |
|  | GLDM | Quantify grey-level dependencies in the tumour | DependenceVariance | Measures variability in how pixel intensities depend on neighbouring pixels, indicating structural heterogeneity. |
|  | NGTDM | quantifies the difference between a gray value and the average gray value of its neighbours within a certain distance | Strength | Measures how clearly structures appear in the image, higher values mean more distinct and coarse patterns. |
|  |  |  | Contrast | Measures local intensity differences, higher values mean more variation between neighboring areas. |

Radiomic features are denoted using the concatenated format ***[image_channel]_***[filter]_***[feature_group]_[feature_name]****.* Image channels include r, g, b, and grey. Filters indicate the preprocessing applied before feature extraction: ***Original*** denotes the unfiltered image; ***Wavelet*** filtering decomposes the image into low-frequency (L) and high-frequency (H) components to capture coarse and fine textural patterns; and ***(LoG)*** filtering (σ = 0.5–5.0 mm) enhances image structures at different spatial scales.

**red = r; green = g; blue = b; greyscale = grey; Laplacian; LoG = *Laplacian of Gaussian*; GLCM** = Grey-Level Co-occurrence Matrix; **GLDM** = Grey-Level Dependence Matrix; **GLSZM** = Grey-Level Size Zone Matrix; **NGTDM** = Neighbouring Grey Tone Difference Matrix; **IMC** = Informational Measure of Correlation; Idmn = Inverse Difference Moment Normalized; MCC = Maximal Correlation Coefficient.

**Section S5. Supplementary figures**


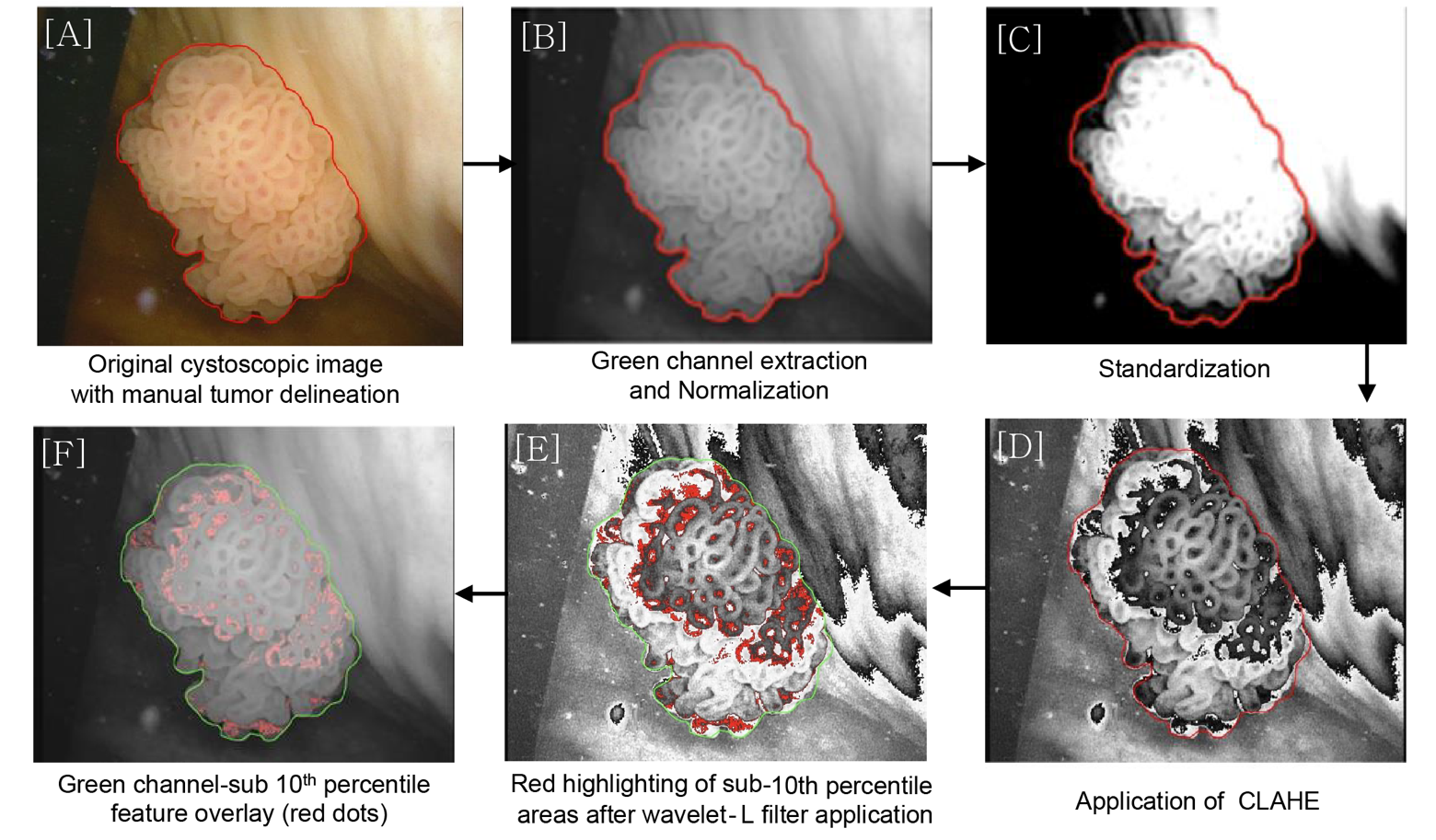


**Supplementary Figure 1.** Systematic preprocessing pipeline for visualisation of the highest-impact radiomics feature (g_wavelet-L_firstorder_10Percentile)

Panels [A–C] show the image preprocessing steps: original cystoscopy image, green‑channel extraction with normalisation, and standardisation. Panels [D–F] show CLAHE and wavelet‑L filtering, followed by overlay of pixels in the lowest 10% of the feature value distribution as red dots within the tumour contour. The model is trained using only the resulting single numerical 10th‑percentile feature value, whereas the red overlays are included solely to qualitatively illustrate the spatial origin of these low‑intensity values for reader interpretability.


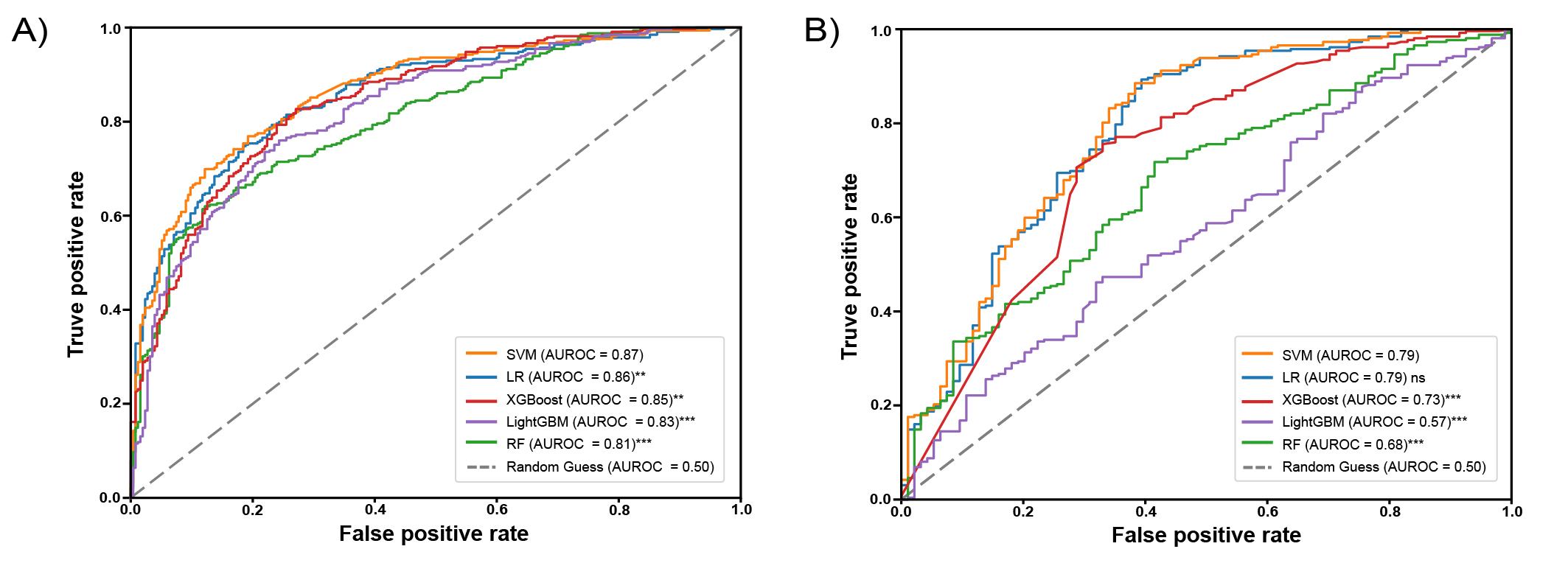


**Supplementary Figure 2.** Performance comparison of radiomics-based classifiers

(**A)** ROC curves of five classifiers (SVM, LR, XGBoost, LightGBM, and RF) evaluated on the internal validation cohort. (**B)** ROC curves of the same five classifiers evaluated on the external validation cohort. Between-model differences in AUROC were assessed using the DeLong test, with statistical significance indicated as follows: ns (P > 0.05), * (P ≤ 0.05), ** (P ≤ 0.01), and *** (P ≤ 0.001).


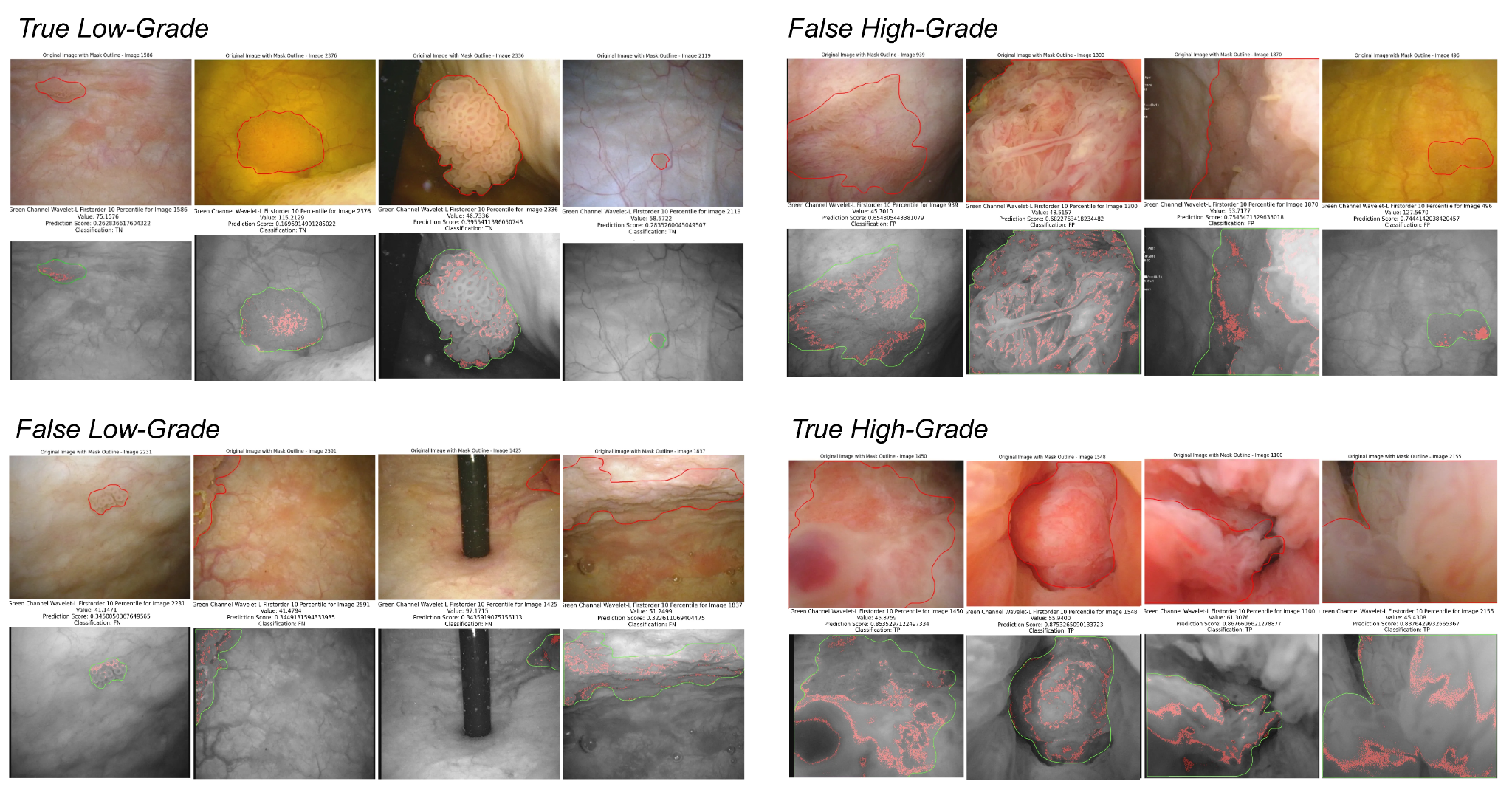


**Supplementary Figure 3.** Analysis of g_wavelet-L First-Order 10th Percentile features between correctly and incorrectly classified bladder cancer grades in the internal validation cohort


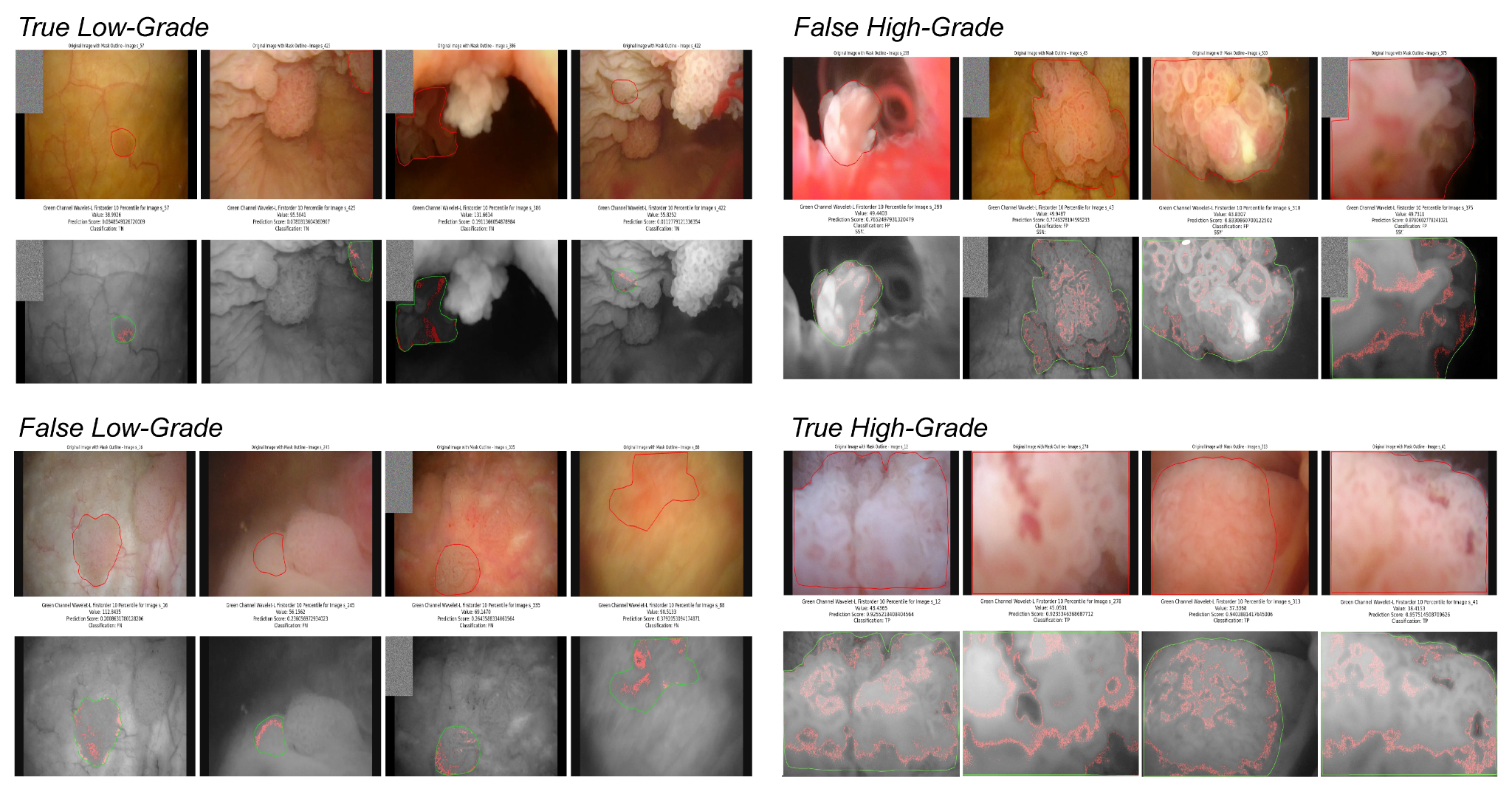


**Supplementary Figure 4.** Analysis of g_wavelet-L First-Order 10th Percentile features between correctly and incorrectly classified bladder cancer grades in the external validation cohort
